# Proximal Tibiofibular Joint Ganglion Cyst: A Rare Cause of Calf Pain

**DOI:** 10.7759/cureus.37810

**Published:** 2023-04-19

**Authors:** Ioannis Papaioannou, Georgia Pantazidou, Vasileios K Mousafeiris, Dimitrios Ntourantonis, Thomas Repantis

**Affiliations:** 1 Orthopedics, General Hospital of Patras, Patras, GRC; 2 Otolaryngology - Head and Neck Surgery, General Hospital of Patras, Patras, GRC; 3 Emergency, University Hospital of Patras, Patras, GRC

**Keywords:** gastrocnemius muscle, pedicle, proximal tibiofibular joint ganglion, intramuscular cyst, knee ganglion cyst

## Abstract

Intramuscular cysts are rare at the proximal calf. However, their etiology is varied, making accurate diagnosis and treatment really difficult. Ganglion cyst (GC) of the proximal tibiofibular (PTF) joint is a very rare entity with an estimated prevalence of 0.76%. Intramuscular extension of the GC arising from the PTF joint is an even rarer lesion, and only a few cases have been published in the literature. Hereby, we report an infrequent case of a GC arising from the PTF joint with a sizable pedicle and intramuscular (lateral head of gastrocnemius) extension to the posterolateral aspect of the right calf.

## Introduction

Cysts around the knee include the well-known Baker’s cysts, meniscal cysts, and cruciate ligament ganglia. Intramuscular cysts of the proximal calf are very rare, and the causes of these cysts are varied. The five types of these cysts are the following: cysts from the knee joint, intramuscular cysts arising from the muscular tissue, neurilemma, cysts from the proximal tibiofibular (PTF) joint, and pigmented villonodular synovitis (PVNS) [1]. Ganglion cyst (GC) of the PTF joint is a very rare entity with an estimated prevalence of just 0.76% [2]. Intramuscular GC of the PTF joint is an even rarer lesion, and only a few cases have been published in the literature. Hereby, a rare case of an intramuscular (lateral head of gastrocnemius) GC in the posterolateral aspect of the right calf arising from the PTF joint with a sizable pedicle is reported.

## Case presentation

**Figure 1:**
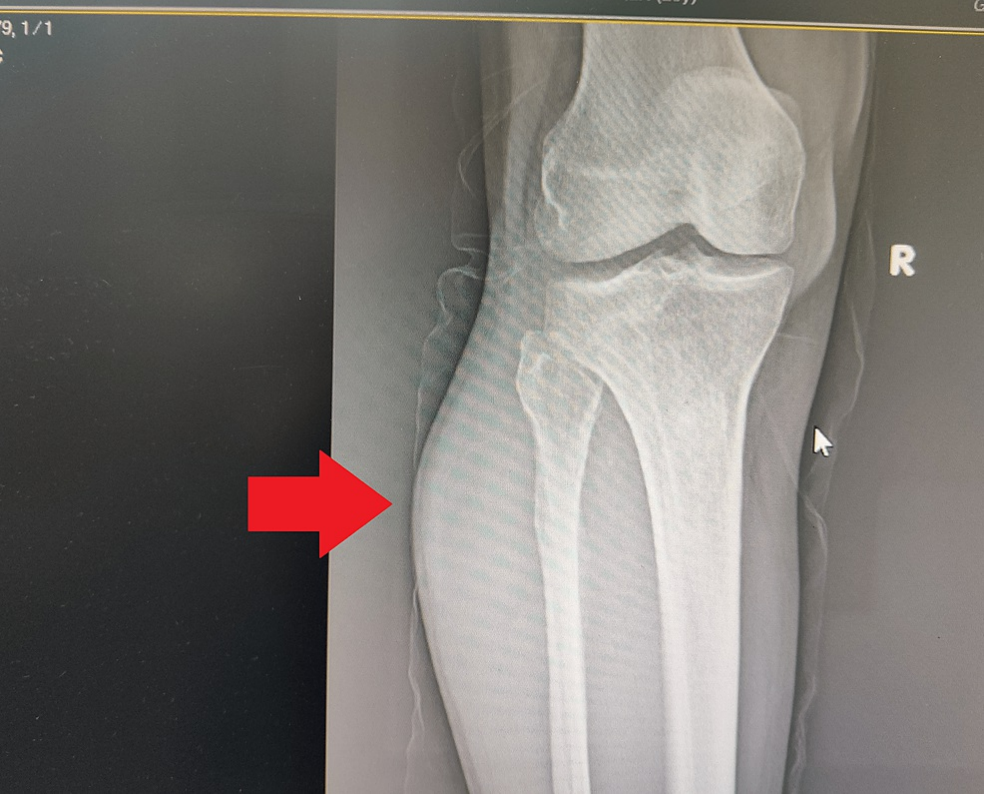
Anteroposterior plain radiograph of the right knee joint demonstrates the bulging of the lateral side of the proximal tibia (red arrow).

A 57-year-old previously healthy male presented with complaints of swelling in the right calf of five months duration. It was not initially associated with any symptoms. However, for two months, he noticed an increase in the size of the swelling and developed an aching pain over the calf, especially during walking and prolonged standing. He denied any history of trauma. Clinical examination revealed an oval cystic swelling of size 7 × 5 cm on the posterolateral aspect of the right leg over the proximal expanse of the gastrocnemius muscle. The swelling was compressible with no obvious involvement of the knee joint on physical examination. There was no evidence of associated neural or vascular compression. Routine laboratory investigations were within normal limits. The anteroposterior plain radiograph demonstrated the bulging of the lateral side of the proximal tibia (Figure 1).

**Figure 2:**
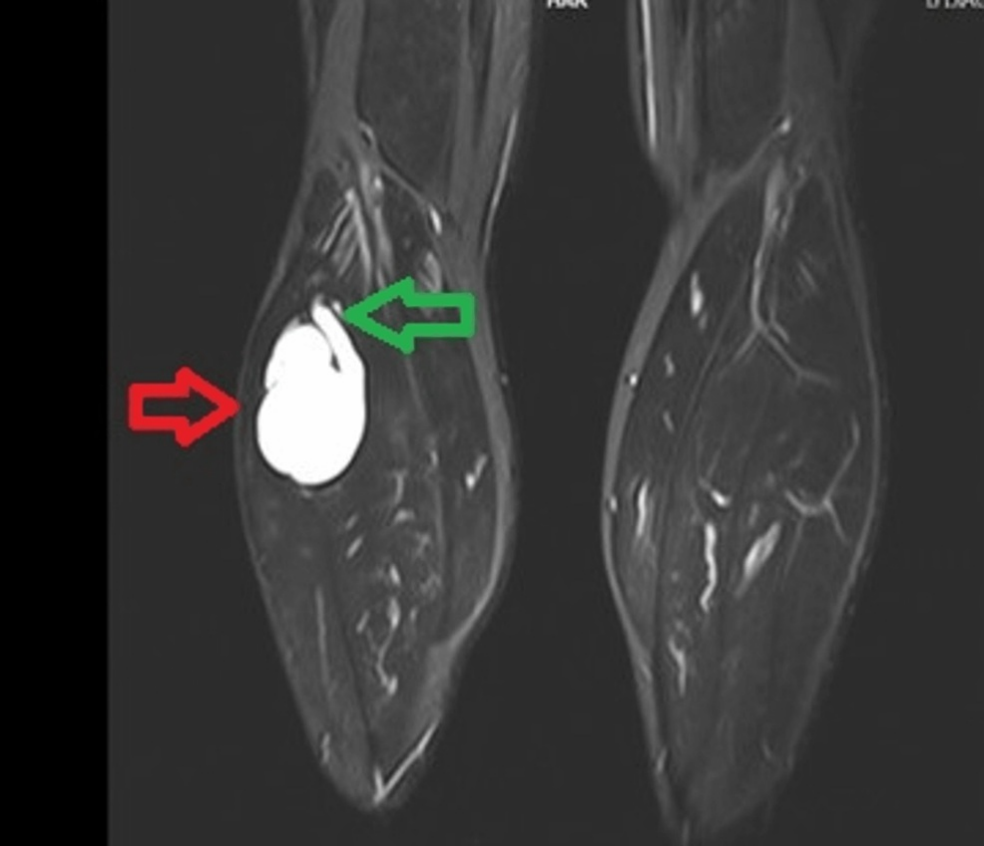
Coronal T2-weighted MRI indicates the lesion (red arrow) and the pedicle (green arrow) arising from the proximal tibiofibular joint. MRI: magnetic resonance imaging

**Figure 3:**
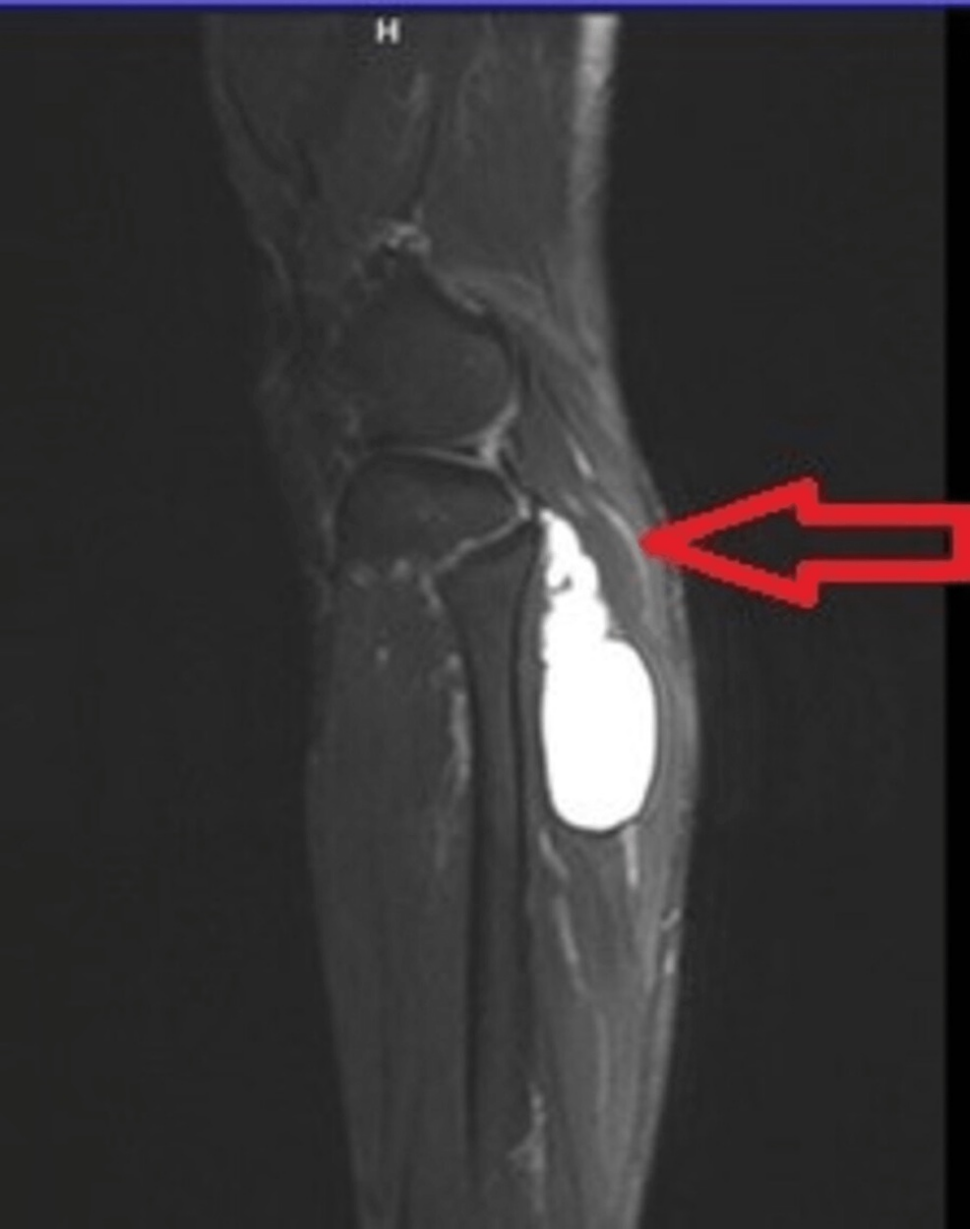
Sagittal T2-weighted MRI indicates the lesion, with the red arrow highlighting the origin of the lesion to the proximal tibiofibular joint. MRI: magnetic resonance imaging

**Figure 4:**
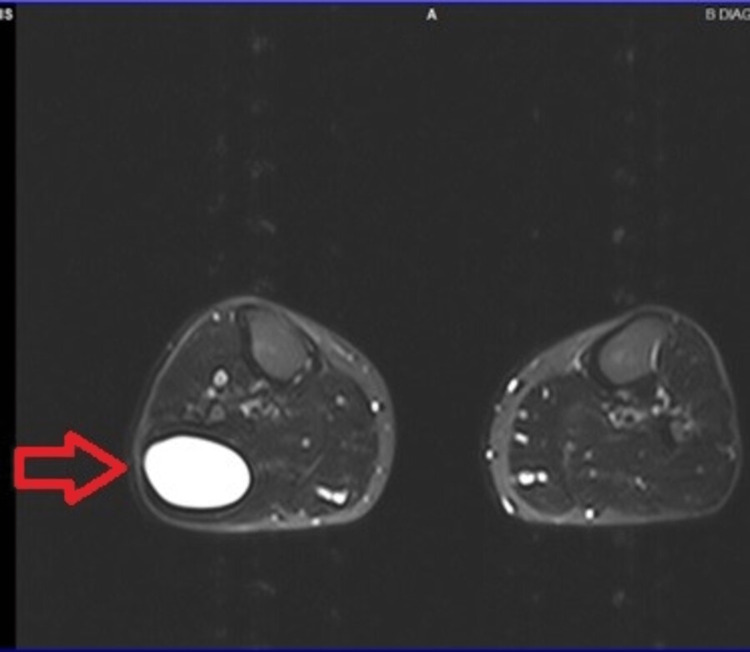
Axial T2-weighted MRI indicates the lesion (red arrow) to the posterolateral aspect of the PTF joint, with cystic form and clear boundaries. MRI: magnetic resonance imaging, PTF: proximal tibiofibular

An ultrasound scan revealed a cystic lesion in the posterolateral aspect of the proximal part of the right calf with extension to the PTF joint. Magnetic resonance imaging (MRI) scan was performed for accurate diagnosis of the lesion, which subsequently revealed a well-defined intramuscular T2-weighted and T2-weighted fat suppression hyperintense cystic lesion, with extension to the posterior aspect of the PTF joint with a pedicle (Figures 2-4).

**Figure 5:**
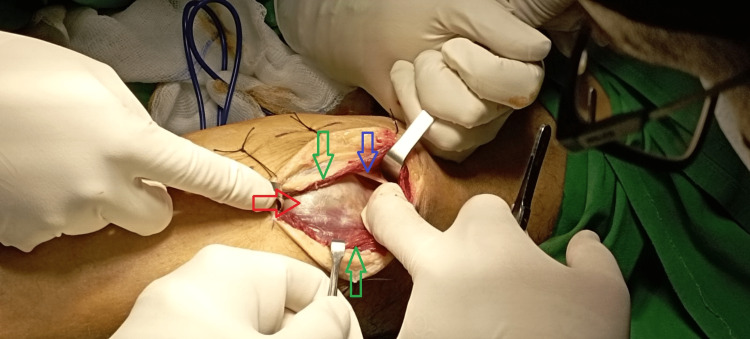
Intraoperative image indicates the location of the lesion (red arrow) under the fascia (blue arrow) within the fibers of the lateral head of the gastrocnemius muscle (green arrows).

The size of the cyst was 7.3 × 4.3 × 3 cm. Based on these findings, the diagnosis of an intramuscular cyst arising from the PTF joint was settled. Surgical excision of the lesion was decided based on our patient’s complaints of pain in the calf, especially in prolonged standing or walking. Complete excision of the cyst was done under general anesthesia and tourniquet usage in the prone position. The surgical incision was done over the lateral gastrocnemius muscle with proximal extension to the posterior aspect of the knee joint in a lazy s fashion. The lesion was found closely adherent within the fibers of the lateral head of the gastrocnemius muscle (Figure 5), while the neurovascular bundle was not recognized as we dissected the posterolateral aspect of the tibia, and we stayed within the gastrocnemius muscle.

**Figure 6:**
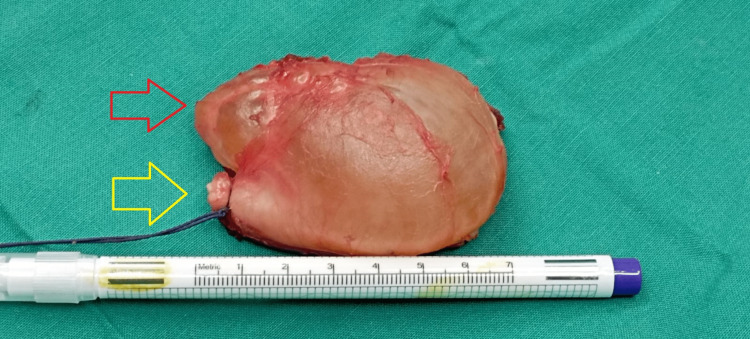
Intraoperative image demonstrates the excised cyst (red arrow), with the yellow arrow indicating the pedicle of the cyst.

**Figure 7:**
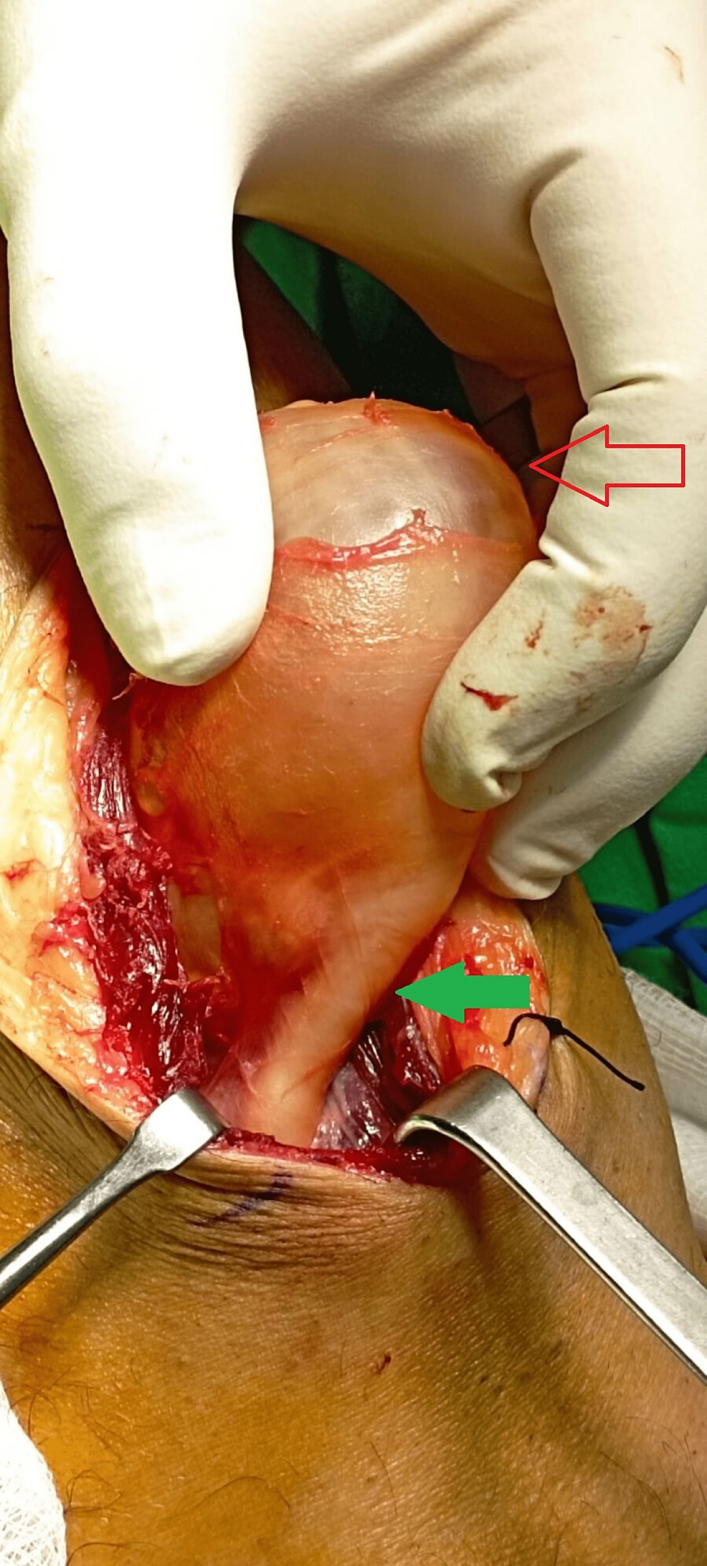
Intraoperative image before the removal of the cyst. The red arrow demonstrates the cyst, while the green arrow indicates the pedicle.

**Figure 8:**
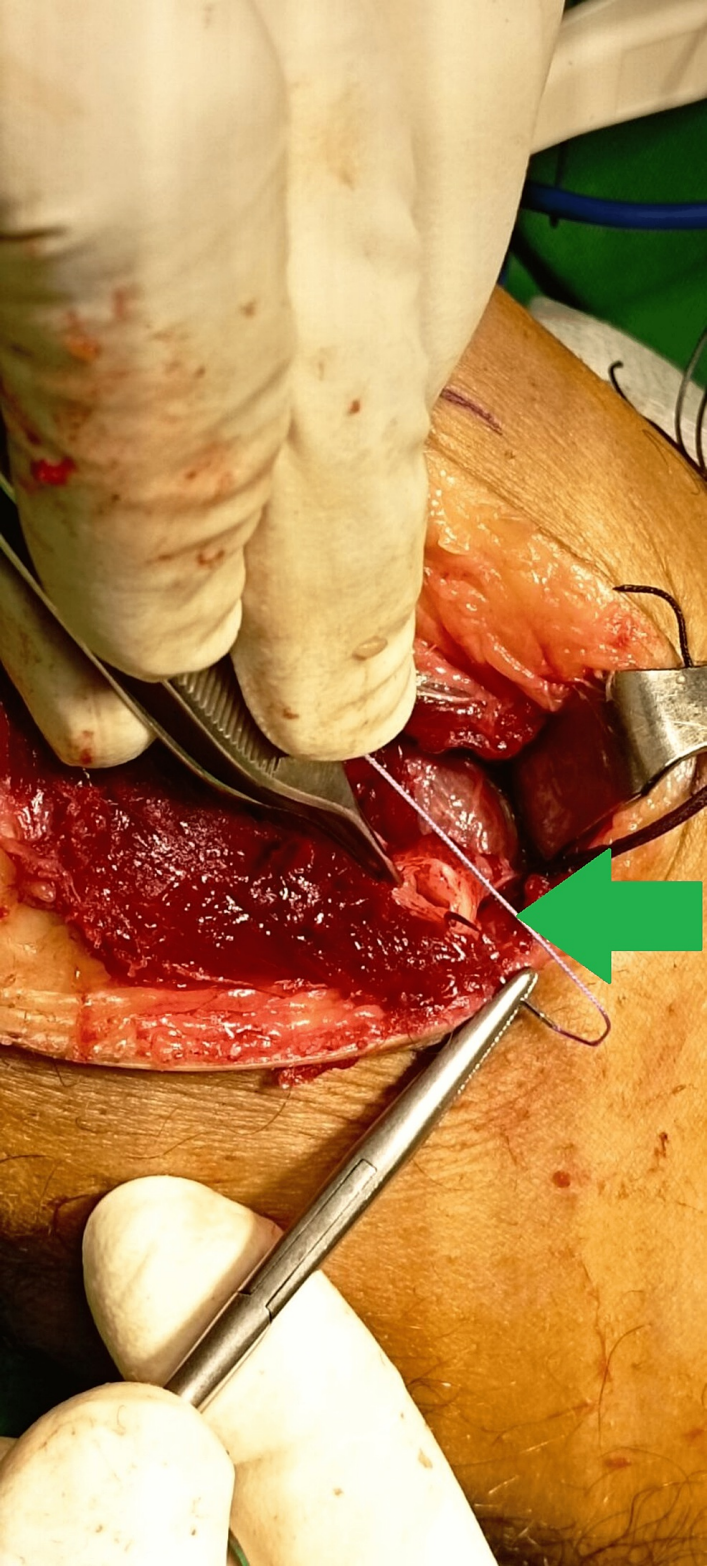
Intraoperative image indicates the remaining pedicle of the cyst (green arrow), which was sutured detailly to avoid any recurrence.

The entire cyst was excised without any rupture of the wall (Figure 6), while the pedicle was recognized and excised from its origin in the posterior aspect of the PTF joint (Figures 7, 8).

**Figure 9:**
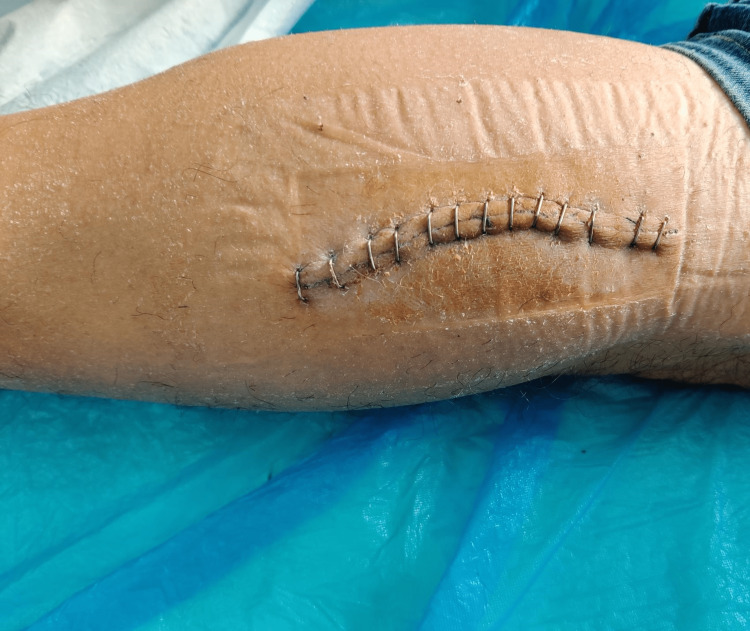
Photo of the calf of the patient 10 days after surgery demonstrates the satisfactory aesthetic result and the reduction of calf swelling due to the cyst.

Histopathological evaluation revealed flattened cells and fibrous tissue, findings consistent with ganglion. The patient had an uneventful recovery and was discharged the next day. The aesthetic result was satisfactory with a subsequent reduction of calf swelling (Figure 9).

The patient was admitted to our outpatient department for the scheduled follow-up at three, six, and 12 months after surgery, and no relapse was recognized.

## Discussion

Intramuscular ganglion cysts arising from the PTF joint at the proximal calf are rare; nevertheless, their etiology is diverse, making accurate diagnosis and treatment really difficult [1]. The most commonly accepted theory is that they generate from secondary myxoid degeneration of connective tissue, which is associated with joint capsules and sheaths of tendons [3]. The treatment of these cysts varies, based on their origin, so it is crucial to identify their derivation. When intramuscular cysts communicate with the PTF joint cavity, they are arising from the PTF joint, as in our case. The clinical presentation of this exact type of cyst is variable, from without symptoms to gradually increasing swelling and pain. Additionally, it is well known that over 50% of cysts of the PTF joint are associated with peroneal nerve palsy, from the compression of the common peroneal nerve in the peroneal canal [4,5]. MRI scan is the gold standard for precise diagnosis [6], while aspiration is not recommended for ganglion cysts as they have a high chance of recurrence [1]. Differential diagnosis includes tendon tear, Baker’s cyst, bursitis, tenosynovitis abscess, myxoma, nerve sheath tumor, vascular lesions, lipomas, and synovial sarcoma [7]. Surgical excision is the gold standard management of ganglion cysts in cases with persistent symptoms and unsuccessful adequate conservative treatment. In addition, surgical intervention of cysts of the PTF joint should be followed by the removal of the pedicle, which connects the cyst to the joint, to reduce the risk of recurrence [5]. To our knowledge, an intramuscular ganglion cyst to the lateral gastrocnemius muscle is a very rare entity with only two cases in the literature [7,8], while an intramuscular ganglion cyst to the lateral gastrocnemius arising from the PTF joint with a huge pedicle has never been reported in the literature. PTF joint intramuscular ganglion cysts are commonly presented in the anterolateral aspect [1] of the knee and frequently are associated with peroneal nerve palsy [1,9]. The most important differential diagnosis in cases with cysts to the posterior aspect of the proximal calf includes Baker’s cyst, although clinicians should be aware that Baker’s cyst usually arises from the semimembranosus-gastrocnemius bursa.

## Conclusions

Intramuscular ganglion cysts arising from the PTF joint are very rare entities. However, clinicians should include this entity in the differential diagnosis of cystic lesions around the knee joint. MRI is the gold standard for a definite diagnosis, while thorough surgical excision accompanied by the removal of the pedicle connected to the cyst to the PTF joint can reduce the chances of relapse. Based on the location of the cyst, symptoms may vary, and not rarely, it can be complicated with peroneal nerve palsy.
